# Cortactin in Epithelial–Mesenchymal Transition

**DOI:** 10.3389/fcell.2020.585619

**Published:** 2020-10-20

**Authors:** Rong Ji, Xiao-Juan Zhu, Zhi-Rong Wang, Li-Qiang Huang

**Affiliations:** Zhangjiagang TCM Hospital Affiliated to Nanjing University of Chinese Medicine, Jiangsu, China

**Keywords:** actin cytoskeleton, cortactin, epithelial-mesenchymal transition (EMT), Arp2/3, N-WASP, ezrin, Snail1, cancer

## Abstract

Cortactin, a member of the actin-binding protein family, plays an important role in cell movement involving the cytoskeleton, as cell movement mediated by cortactin may induce the epithelial–mesenchymal transition. Cortactin participates in tumor proliferation, migration, and invasion and other related disease processes by binding to different proteins and participating in different pathways and mechanisms that induce the occurrence of these disease processes. Therefore, this article reviews the correlations between cortactin, the actin cytoskeleton, and the epithelial–mesenchymal transition and discusses its clinical importance in tumor therapy.

## Introduction

The internal cytoskeleton of eukaryotic cells is composed of actin filaments, microtubules, and intermediate filaments (Remedios et al., 2003). As the main component of microfilaments, actin forms actin filaments that interact with numerous accessory proteins to generate the actin cytoskeleton (Svitkina, 2018). Actin filaments are formed by the polymerization of spherical monomeric actin (G-actin) with a polar structure into the double helical structure of filamentous actin (F-actin) with two different ends (Feldt et al., 2019). The actin cytoskeleton is the primary cellular machinery that generates force. It produces pushing, pulling, and resistance forces. These forces are produced by the coordinated polymerization of various actin filaments, sliding of bipolar filaments of myosin II along actin filaments, and the formation of a cross-linked membrane-related filament array, respectively (Svitkina, 2018). The force produced by the actin cytoskeleton is important for many cell movements, the structure and mechanical energy of the cytoplasmic matrix (Pollard and Cooper, 1986), and facilitates embryonic development, immune defenses, and wound healing (Kirkbride et al., 2011). The movement of the actin cytoskeleton in cells is mainly promoted by the binding of actin and corresponding proteins (Feldt et al., 2019). These proteins are called actin-binding proteins (ABPs). To date, 162 ABPs have been discovered. ABPs include membrane-associated proteins, membrane receptors, and ion transporters. These proteins are involved in the cross-linking of actin filaments, mediate the interactions of microfilaments with other cytoskeletal components, and promote the polymerization and depolymerization of filaments (Qu et al., 2007).

Cortactin, a member of the ABP family, plays an important role in cell movement involving the cytoskeleton (Zhang et al., 2009). Cell movement mediated by cortactin induces the epithelial–mesenchymal transition (EMT) and then participates in relevant disease processes, such as tumor proliferation, migration, and invasion (Karamanou et al., 2020). The inhibition of cortactin blocks the EMT, thereby preventing the proliferation, migration, and invasion of cancer cells (Huang et al., 2019). From a clinical perspective, these results support the application of cortactin as a promising therapeutic target for diseases such as cancer (Yilmaz and Christofori, 2009; Yin et al., 2017). Therefore, this article reviews the correlations between cortactin, the actin cytoskeleton, and the EMT and discusses its clinical value in tumor therapy.

### Cortactin

**FIGURE 1:**
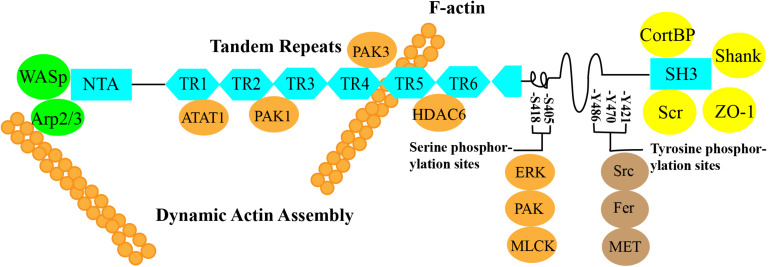
➀ N-terminal acidic region (NTA): The NTA of cortactin binds to Arp2/3 alone or cooperates with Wiskott–Aldrich syndrome protein (WASP) to bind Arp2/3 to regulate branched actin assembly and regulate F-actin polymerization and contraction (Weed et al., 2000; Takehito et al., 2001; Weaver et al., 2002; Pant et al., 2006). ➁ Cortactin function can be regulated by posttranslational modifications of the 6.5 F-actin repeat domain, which contains a repeating 37-amino acid sequence. These modifications include phosphorylation and acetylation by PAK1, PAK3, ATAT1, and HDAC6 (Ohoka and Takai, 1998; van Rossum et al., 2003, 2005; Katsube et al., 2004; Zhang et al., 2007; Castro-Castro et al., 2012; Hayes et al., 2013; Li et al., 2017). ➂ SH3: The C-terminal domain allows actin to function as a scaffold protein because many cytoskeletal, membrane transport, and signaling proteins are bound to the C-terminal SH3 domain, such as ZO-1, CortBP, and Shank (Weed et al., 1998; Weed and Parsons, 2001; Burkhardt et al., 2008). ➃ The central part of the protein between the F-actin repeat domain and the SH3 domain contains an α-helix and a proline-rich region that includes three tyrosine phosphorylation sites, Y421, Y470, and Y486 (human), which are phosphorylated by Src, Fer, and c-Met. The two serine phosphorylation sites, S405 and S418, are phosphorylated by ERK, PAK, MLCK, and other kinases (Beaty et al., 2013; Rosse et al., 2014; Moshfegh et al., 2015).

Cortactin is an F-actin-binding protein that regulates cell movement and adhesion junction assembly (Zhang et al., 2009). Cortactin is located on chromosome 11q13, is composed of 550 amino acids, and has a molecular weight of 61.582 kDa (Schuuring et al., 1993). According to the amino acid sequence of cortactin, it contains three important structural domains: an N-terminal acidic region (NTA), a 6.5 F-actin repeat structural domain, and a C-terminal SH3 structural domain. An α-helix and a proline-rich region are located in the central part of the protein between the F-actin repeat structural domain and the SH3 structural domain (Figure 1).

### Cortactin and the Actin Cytoskeleton

Cortactin is a primary regulator of the actin cytoskeleton. At a specific time and in discrete places within the cell, cortactin binding to F-actin regulates the structure of the actin cytoskeleton, thereby altering the morphology and function of the cell (Stossel et al., 1985). It also regulates the formation of corpuscles and lamellipodia, integrin signaling, axon guidance, and extracellular matrix degradation (Yamaguchi and Condeelis, 2007; Weaver, 2008; Marioni et al., 2018). The expression and cytoplasmic localization of cortactin are also crucial for maintaining the structure of the actin cytoskeleton (Motonishi et al., 2015). Overexpression of cortactin increases cell viability (Hill et al., 2006; Rothschild et al., 2006), mainly because of its functions in the assembly of the actin cytoskeleton and in promoting the persistence of lamellar protrusions (Bryce et al., 2005). When cortactin is located at the margin of the cell, it regulates the structure of the actin cytoskeleton and promotes the formation of an invasive pseudopod, which plays a complex role in the EMT, promotes the *in situ* polymerization of actin, and regulates autocrine signaling (Wang et al., 2013). Therefore, all cell activities in which cortactin is involved, including cell migration, invasion, and localization, require the actin cytoskeleton (Weed et al., 2000; Uruno et al., 2001; Katsube et al., 2004; Ayala et al., 2008).

### The EMT and the Actin Cytoskeleton

The EMT was first discovered in embryonic cells (Hugo et al., 2007). According to a recent study, the EMT occurs naturally in numerous tissue types and various stages of development (Zhang and Weinberg, 2018). The EMT is crucial for normal development and tissue remodeling and contributes to disease progression, e.g., fibrosis and cancer metastasis (Kalluri and Neilson, 2003; Borok et al., 2011; Rana et al., 2018). The mechanism of the EMT is to convert epithelial cells into cells with a mesenchymal phenotype that are arranged along the epithelial and mesenchymal axes (Zhang and Weinberg, 2018). Epithelial cells exhibit interepithelial cell connections and apical bases, whereas mesenchymal cells exhibit increased motility and invasiveness and lack a spindle-like morphology and basic polarity (Nieto et al., 2016). Therefore, the occurrence of the EMT plays an important role in cell transformation. The EMT is the initial stage and necessary step of the transfer cascade reaction and is characterized by the loss of root tip polarity and intercellular adhesion, and the morphology and movement of mesenchymal cells occur through cytoskeletal reconstruction (Yilmaz and Christofori, 2009; Tsai et al., 2012; Nieto, 2013; Ye et al., 2016). Cytoskeletal reconstruction is based on the balance and control of the extent of the local assembly and disassembly of actin filaments (Yilmaz and Christofori, 2009). During the EMT, the cytoskeleton must be reshaped at the leading edge to form pseudopodia and allow the cell to move in the surrounding environment (Huang et al., 2019). Therefore, cytoskeletal reconstruction is crucial in the process of EMT-induced cell transformation.

### Proteins That Interact With Cortactin

**FIGURE 2:**
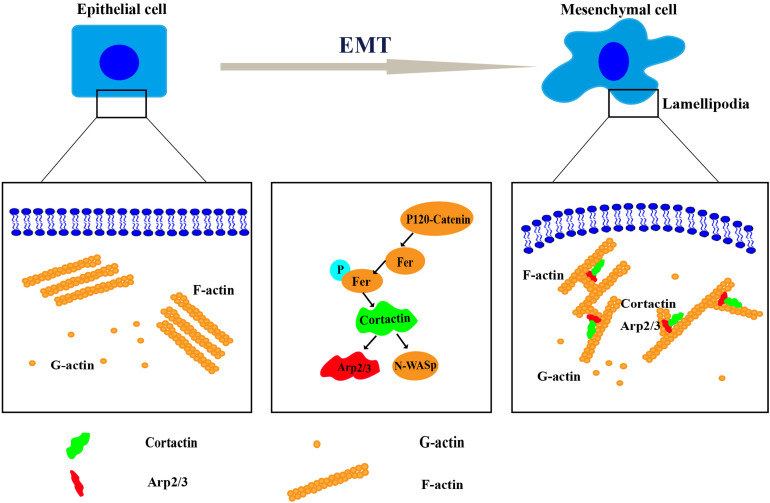
The mechanism of cortactin-induced cytoskeletal remodeling during the epithelial–mesenchymal transition (EMT). Remodeling of the cytoskeleton requires the coordination of several processes, including the protrusion of a lamellipodium at the leading edge, adhesion, contraction of the actin bundle, and retraction of the trailing edge of the cell. Cortactin regulates cytoskeletal remodeling by interacting with Arp2/3, neural Wiskott–Aldrich syndrome protein (N-WASP), and other proteins. Then, it promotes the EMT.

Cortactin, a key regulator of actin cytoskeletal assembly and remodeling, is mainly distributed in structures required for cell movement, such as lamellipodia and filopodia. Cortactin-induced dynamic reconstruction of the actin cytoskeleton provides the motile force that promotes the occurrence of the EMT (Huang et al., 2019). Cortactin regulated the occurrence of the EMT through various mechanisms, such as synergy with *E*-cadherin to induce the occurrence of the EMT (Yilmaz and Christofori, 2009), a change in cortactin expression that reshapes the actin cytoskeleton and induces the invasion of single cells and groups of cells (Yamaguchi and Condeelis, 2007), and the mutual effects of cortactin and site-specific binding partners on inducing different cellular activities, including the EMT (Helwani et al., 2004; Kirkbride et al., 2011) (Figure 2).

#### Cortactin and Arp2/3

The Arp2/3 complex is the primary molecular regulator of actin polymerization and is essential for the nucleation and formation of branched actin filament networks in cells (Pollard et al., 2000; Luo, 2002). The branched actin network provides the cellular structure and facilitates processes involving the plasma membrane, such as the formation of the cell–cell connection formation (Johnston et al., 2008), the motility of pathogens, the transport of vesicles, and the formation of cell–cell junctions (Goley and Welch, 2006; Garcia-Ponce et al., 2015). Cellular signals activate the inactive Arp2/3 complex (Higgs and Pollard, 1999). As a scaffold protein and activator of the Arp2/3 complex, cortactin interacts with and activates the Arp2/3 complex through its NTA (Higgs et al., 1999; Machesky et al., 1999; Yin et al., 2017). The cortactin and Arp2/3 complex is colocalized on dynamic particle structures rich in actin filaments. Through this biological process, actin filaments are assembled and extend to the nucleus to form a protein filament network, where the actin filament branch is stimulated (Takehito et al., 2001; Weaver et al., 2001). Cortactin mainly promotes the formation of the shape of the branched actin network by the Arp2/3 complex through two mechanisms. First, cortactin activates Arp2/3 separately and interacts with N-Wiskott–Aldrich syndrome protein (WASP), after which cortactin inhibits the disintegration of the preformed Arp2/3-core filament network (Weaver et al., 2001). The activated Arp2/3 complex produces unbranched actin filaments and branched actin filaments to induce the EMT (Rana et al., 2018).

#### Cortactin and N-WASP

Members of the WASP family, such as N-WASP, participate in the mechanism regulating the reorganization of the actin cytoskeletal and induce the occurrence of the EMT (Salvi and Thanabalu, 2017). The EMT induced by N-WASP mainly results in the reconstruction of the actin cytoskeleton through the activation of the Arp2/3 complex. However, N-WASP and cortactin activate the Arp2/3 complex alone or in combination (Helgeson et al., 2014). Both cortactin and N-WASP contain an acidic structural domain, which is necessary for binding to the Arp2/3 complex (Weaver et al., 2002). Phosphorylation at specific sites regulates the activities of cortactin and N-WASP, which are two important regulators of actin nucleation (Uzair et al., 2019). When cortactin is phosphorylated and overexpressed in cells, it interacts with N-WASP to facilitate actin polymerization (Wu and Parsons, 1993; Luttrell et al., 1994; Artym et al., 2006; Tehrani et al., 2007; Oser et al., 2009). For instance, the phosphorylation of cortactin by the serine/threonine kinase extracellular regulatory kinase 1/2 and p21-activated kinase 1 enhances the interaction of N-WASP with cortactin (Martinez-Quiles et al., 2004; Grassart et al., 2010) and promotes its phosphorylation of the Arp2/3 complex and transport to the plasmalemma to induce the EMT (Uzair et al., 2019).

#### Cortactin and Ezrin

Ezrin is a crosslinker of the actin skeleton and cell membrane, and a member of the ezrin–radixin–moesin family (He et al., 2017). Ezrin interacts with cortactin to induce various cellular processes, such as the regulation of the assembly of branched actin filaments, cell–cell adhesion, membrane transport, and ECM degradation (Sung et al., 2011). Both ezrin and cortactin are closely related to cell migration, which is an important component of the EMT process (He et al., 2017). Moreover, phosphorylation has been shown to enhance the function of cortactin by changing the complement of cortactin-bound proteins during migration and invasion (Lapetina et al., 2009; Oser et al., 2009, 2010; Kelley et al., 2010), thereby promoting the invasion and metastasis of various tumors (Weaver, 2008; Ni et al., 2015; Li et al., 2016). The interaction of ezrin with cortactin is a new mechanism involved in the EMT during the tumor metastasis process (He et al., 2017).

#### Cortactin and Snail1

Snail1 and cortactin are the key factors among all EMT-related proteins (Wu et al., 2015). Cortactin plays important roles in cellular migration and endocytosis, and Snail1 is a potential EMT activator that directly inhibits *E*-cadherin. Snail1 regulates proteins containing an E-box motif, including *E*-cadherin, in various tumors as a transcriptional repressor (Cano et al., 2000). This Snail1-mediated inhibition of *E*-cadherin is generally considered one of the signs of the EMT. During tumor metastasis, the EMT transforms epithelial cells into active and aggressive mesenchymal cells (Villarejo et al., 2014). Snail1 and cortactin both promote the EMT; however, recent reports have identified a negative regulatory effect of Snail1 on cortactin (Lee et al., 2014). For instance, during cultivation in a 3D collagen gel, the exposure of MDA-MB-231 cells to different environmental stimuli increases Snail1 expression and reduces cortactin expression. Inhibition of the JNK signaling pathway increases the expression of Snail1, which subsequently inhibits cortactin, thereby regulating the occurrence of the EMT (Lee et al., 2014).

### Participating Pathways

Cortactin is the main organizer of membranous and invasive protrusions and is a presynaptic regulator of rapid activity-dependent signaling in synaptic structures. It induces the EMT by participating in multiple pathways. The level of cortactin at the membrane of the stimulated synapse is increased, which requires neuronal activity, *de novo* transcription, and Wg/Wnt-dependent expression (Alicea et al., 2017). In melanoma, RNF128 interferes with the ubiquitination and degradation of CD44 and cortactin proteins, activates the Wnt pathway, and promotes the cellular EMT and stem cell development (Wei et al., 2019). Cortactin also induces the EMT by participating in the pathway regulated by the Rho GTPase Rac1. Local actin polymerization is induced by mechanical stimuli and *N*-cadherin, therefore ensuring the integrity of the adhesion complex. The Rho GTPase Rac1 is activated by *N*-cadherin, which recruits cortactin to the *N*-cadherin adhesion complex (El Sayegh et al., 2005). In this process, Fer, a non-receptor tyrosine kinase, binds to N-cadherin through a mechanism mediated by p120-catenin (Kim and Wong, 1995). The phosphorylation of Fer activates cortactin, which induces the reorganization of the actin cytoskeleton, increases the movement of *N*-cadherin, extends the adhesion area, and then promotes the formation of a stable cell adhesion to ultimately regulate the shape of the cell. In addition to the aforementioned pathways, cortactin also participates in other pathways to induce the EMT. The related pathways and upstream and downstream molecules are shown in Table 1.

**TABLE 1:** Correlated signaling pathways and upstream and downstream molecules related to cortactin-induced epithelial–mesenchymal transition (EMT).

**Pathways**	**Upstream**	**Downstream**	**References**
Rac1/PAK1/cortactin	P27, PAK1	ECM	Jeannot et al., 2017
Wg/Wnt signaling pathway	RNF128	CD44, c-Myc	Alicea et al., 2017; Wei et al., 2019
Rho GTPase Rac1 signaling Pathway	PODXL, CD44	FAK, Paxillin	Kim and Wong, 1995; El Sayegh et al., 2005
T3αvβ3 FAK/paxillin/cortactin/NWASP Arp2/3 signaling pathway	T3, FAK, Paxillin	NWASP, Arp2/3	Uzair et al., 2019
FAK/Src signaling pathway	Scr, P53	FAK, F-actin	Wang et al., 2013; Ungewiss et al., 2016
EGFR/Src/Arg/cortactin signaling pathway	Src, Arg, EGF	ECM	Mader et al., 2011; Ni et al., 2015

### Cortactin in Cancer

Cortactin is overexpressed in many epithelial cancers (Marioni et al., 2017). In human tumors, cortactin overexpression leads to increased cell migration, invasion, and metastasis (Clark and Weaver, 2008). The transformation of breast cancer is related to the EMT. During this process, actin-rich protrusions on the serosa form invasive pseudopodia, which promote protein degradation in the extracellular matrix and tumor invasion. The formation and main function of invasive pseudopodia are controlled by cortactin (Karamanou et al., 2020). The polymerization of phosphorylated cortactin and actin at aggressive pseudopodia increases the invasiveness of human breast cancer cells and subsequently induces matrix degradation and aggressive behavior (Mader et al., 2011). In pancreatic ductal adenocarcinoma, the overexpression and phosphorylation of cortactin promotes the occurrence of the EMT, followed by the metastasis and migration of pancreatic ductal carcinoma (Stock et al., 2019). Cortactin is mainly phosphorylated at Y421 (Head et al., 2003), resulting in increased recruitment of proteins containing an SH2 domain, activation of the Arp2/3 complex, and increased stability and conversion of focal adhesions (Okamura and Resh, 1995; Tehrani et al., 2007). The tyrosine phosphorylation of cortactin is often used as an EMT marker due to its relationship with the protease activity necessarily for matrix degradation and cell invasion (Bowden et al., 2006). Cortactin induces the EMT in other types of cancer and promotes tumorigenesis (Table 2).

**TABLE 2:** Cortactin induces EMT in other cancer syndromes and promotes the development of cancer syndromes.

**Disease**	**The expression of cortactin**	**Related mechanism**	**References**
Oral squamous cell carcinoma	Increased phosphorylation of cortactin	In SCC-15_Tβ4 and SCC-25_Tβ4 cells, EMT-induced transcription factors were significantly enhanced. Overexpression of Tβ4 increased the *in vitro* invasion and MMP-2 activity and enhanced the phosphorylation of paxillin and cortactin and the expression of LIMK1.	Hong et al., 2016
Melanoma	Cortactin ubiquitination and degradation	The downregulation of RNF128 promotes the progression of melanoma by ubiquitination and degradation of CD44/cortactin to activate Wnt signaling, thereby inducing cellular EMT and obtaining stem cells.	Wei et al., 2019
Gastric carcinoma	Cortactin overexpression	Cortactin can promote the proliferation of gastric cancer cells and EMT, while microRNA-545 (miR-545) can inhibit the expression levels of cortactin mRNA and protein in GC cells and has a negative regulatory effect on the carcinogenic activity of cortactin.	Ma et al., 2018

## Conclusion and Prospects

Based on accumulating evidence from recent studies, cortactin is an important ABP. Its binding to F-actin regulates cell movement and adhesion. Cortactin also regulates the structure of the actin cytoskeleton by binding to various protein, thereby inducing the occurrence of the EMT. The role of the EMT in the occurrence and development of diseases, particularly its important role in tumor metastasis, has gradually attracted attention. Therefore, this article discusses the role of cortactin in the induction of the EMT by binding to different proteins and participating in different pathways and mechanisms to induce the occurrence of the EMT. However, other related binding proteins and mechanisms of cortactin in the EMT remain to be discovered. Perhaps we will be able to prevent the development of diseases by regulating the activity of cortactin-related proteins and pathways to control the EMT. As researchers are increasingly focusing on the role of cortactin complexed with different proteins in diseases, additional regulatory mechanisms will be discovered. The mechanisms will provide new insights for studies of related diseases and the development of new drugs and treatment methods and will provide additional evidence for the clinical application of cortactin in the future.

## Author Contributions

L-QH designed the work. RJ and X-JZ wrote the manuscript and prepared the figures. Z-RW drafted and revised the manuscript. All the authors contributed to manuscript revision, read and approved the submitted version.

## Conflict of Interest

The authors declare that the research was conducted in the absence of any commercial or financial relationships that could be construed as a potential conflict of interest.
